# Interventions to improve work outcomes in work-related PTSD: a systematic review

**DOI:** 10.1186/1471-2458-11-838

**Published:** 2011-10-31

**Authors:** Erene Stergiopoulos, Adriana Cimo, Chiachen Cheng, Sarah Bonato, Carolyn S Dewa

**Affiliations:** 1 Centre for Research on Employment and Workplace Health, Centre for Addition and Mental Health, 455 Spadina Avenue, Suite 300, Toronto, M5S 2G8, Canada; 2 Canadian Mental Health Association, Clinic & Resource Centre, 272 Park Avenue, Thunder Bay, P7B 1C5, Canada; 3 Library Services, Centre for Addiction and Mental Health, 33 Russell Street, Toronto, M5S 2S1, Canada; 4 Department of Psychiatry, University of Toronto, 250 College Street, Toronto, M5T 1R8, Canada

## Abstract

**Background:**

Posttraumatic stress disorder acquired at work can be debilitating both for workers and their employers. The disorder can result in increased sick leave, reduced productivity, and even unemployment. Furthermore, workers are especially unlikely to return to their previous place of employment after a traumatic incident at work because of the traumatic memories and symptoms of avoidance that typically accompany the disorder. Therefore, intervening in work-related PTSD becomes especially important in order to get workers back to the workplace.

**Methods:**

A systematic literature search was conducted using Medline, PsycINFO, Embase, and Web of Science. The articles were independently screened based on inclusion and exclusion criteria, followed by a quality assessment of all included articles.

**Results:**

The systematic search identified seven articles for inclusion in the review. These consisted of six research articles and one systematic review. The review focused specifically on interventions using real exposure techniques for anxiety disorders in the workplace. In the research articles addressed in the current review, study populations included police officers, public transportation workers, and employees injured at work. The studies examined the effectiveness of EMDR, cognitive-behavioural techniques, and an integrative therapy approach called brief eclectic psychotherapy. Interestingly, 2 of the 6 research articles addressed add-on treatments for workplace PTSD, which were designed to treat workers with PTSD who failed to respond to traditional evidence-based psychotherapy.

**Conclusions:**

Results of the current review suggest that work-related interventions show promise as effective strategies for promoting return to work in employees who acquired PTSD in the workplace. Further research is needed in this area to determine how different occupational groups with specific types of traumatic exposure might respond differently to work-tailored treatments.

## Background

The most recent practice guidelines from the International Society for Traumatic Stress Studies suggest that posttraumatic stress disorder (PTSD) in the workplace may be a common problem, though there has been little research in the area [1]. Indeed, PTSD results in approximately 3.6 lost work days every month in the United States, which is comparable to the work impairment related to major depression [2]. PTSD can develop following a traumatic event, which is a situation involving the threat of death or serious injury to oneself or others [3]. Workers are a relatively understudied subpopulation of PTSD sufferers, yet their risk of facing traumatic situations is well-documented. For instance, employees in emergency services (i.e., firefighters, police officers, and rescue workers) encounter violence and accidents on a frequent basis [4,5]. Workers in factory facilities face the risk of injury from accident, and those in the services industry (i.e. store clerks, bank tellers) might experience violent incidents or robberies at work [6].

Since a key symptom of PTSD involves avoiding environments related to the traumatic event, employees traumatized at work often have extreme difficulty returning to their place of employment [7]. However, employees who are not able to return to work also experience more persistent PTSD symptoms [8]. Workers are therefore left in a vicious cycle, where their PTSD symptoms keep them from working, and their absence from work keeps them from overcoming the disorder.

Traumatized employees are not the only ones to suffer the burden of PTSD -- which includes vivid flashbacks to the traumatic event, difficulty sleeping and concentrating, and excessive vigilance. Employers in turn face the negative consequences of absenteeism and decreased productivity [9,2,10]. Considering the harmful effects of PTSD in the workplace, there is a clear need for interventions to rehabilitate employees, both by eliminating their symptoms and helping them return to work.

One recent review by Nordik et al. (2010) assessed the effectiveness of interventions for anxiety disorders in the workplace [11]. Their review included two studies of PTSD, and focused specifically on interventions involving real-life exposure to environments or objects related to the traumatic event. However, by focusing exclusively on these types of interventions, their article did not address the full range of evidence-based treatments. Therefore, the present study aims to extend their review by examining the most effective interventions for work-related PTSD, in order to inform the decisions of employers, employees, and healthcare providers.

### PTSD symptoms and diagnosis

PTSD is an anxiety disorder characterized by psychological and physical symptoms following a catastrophic life event [12]. Individuals can experience trauma when they face the threat of death or serious injury to themselves or others [3]. The diagnostic criteria of PTSD, according to the DSM-IV-TR, include three major groups of symptoms. First, people with PTSD suffer from re-experiencing of the traumatic event. This can include unwanted flashbacks, dreams, or distress following reminders of the trauma. Second, people with PTSD demonstrate avoidance of environments or objects linked with the trauma, as well as emotional numbing. Symptoms in this category can include feelings of detachment, diminished interest in usual activities, and blunted emotions. Finally, they show increased signs of physical stress, which includes difficulty falling or staying asleep, irritability, concentration difficulties, or an exaggerated startle response [12].

### PTSD treatment approaches

A wide variety of treatment options are available for people with PTSD. Psychotherapy is a form of treatment involving counseling from a trained therapist, and does not include medications. It is also a well-established intervention for clients with PTSD [13]. Psychotherapy itself encompasses a range of treatments. For instance, cognitive behaviour therapy (CBT) focuses on eliminating clients' negative beliefs about themselves while gradually exposing them to the thoughts and situations they are afraid of [14]. Eye movement desensitization and reprocessing (EMDR) is a similar manual-based treatment that incorporates methods from CBT, as well as a "bilateral stimulation" component -- during which clients move their eyes back and forth as they recall both negative and positive memories [15]. Psychodynamic psychotherapy for PTSD focuses on clients' unconscious conflicts from childhood onward, with a larger emphasis on their relationship with the therapist [16]. Please refer to Table 1 for detailed definitions of each psychotherapy treatment used for PTSD.

**Table 1:** Summary of psychotherapeutic treatments for PTSD

Treatment	Description
Cognitive behaviour therapy (CBT)	Helps challenge clients' negative beliefs about their ability to cope with stressful situations. Gradually exposes them to feared situations and objects in order to eliminate the fear.

Eye movement desensitization and reprocessing (EMDR)	Incorporates methods from CBT and other therapies to help clients revisit and reprocess traumatic memories so they become less distressing.

Psychodynamic psychotherapy	Focuses on clients' unconscious and unresolved conflicts, and how these might contribute to current symptoms. Emphasizes the clients' relationship with the therapist.

Hypnotherapy	A form of psychotherapy that uses hypnosis.

A number of meta-analyses have found that CBT and EMDR in particular are the most efficacious psychotherapies for treating PTSD among general civilian and veteran samples. Bradley et al. (2005) found that CBT and EMDR have similar effects on symptom reduction, and Bisson et al. (2007) found no difference in efficacy between trauma-focused CBT and EMDR [17,18]. Both of these reviews pointed to the effectiveness of CBT and EMDR over other treatments, such as stress management, psychodynamic psychotherapy, and waitlist control condition. The most recent meta-analysis by Benish et al. (2008) contends that there are in fact no differences in efficacy among what they call "bona fide" PTSD treatments [13]. The authors define "bona fide" treatments as those that are "intended to be therapeutic" (pp. 748) [13]. In their analysis, such treatments included EMDR, hypnotherapy, psychodynamic psychotherapy, and various forms of cognitive-behavioural interventions. The success of these strategies points to the high level of success found for psychotherapy targeted at PTSD. The purpose of this review is therefore to examine the evidence for the effectiveness of existing interventions adapted for work-related PTSD.

## Methods

### Literature search

This paper was based on a systematic literature search conducted on June 18, 2011 using Medline, PsycINFO, Embase, and ISI Web of Science databases. Thus, it did not use any primary data nor did it involve any animal or human subjects. The search strategies, presented in Appendix 1, were developed in consultation with a library scientist (SB). Raters ES and AC independently screened titles, abstracts, and full-text articles resulting from the search with criteria developed and piloted *a priori*. A review of the Cochrane Occupational Safety and Health Review Database did not yield any relevant results, and databases specific to occupational health, such as NIOSH, were found to lack reports of psychological functioning at work. Key journals indexed by these databases that did address psychological issues at work were also indexed by other databases included in the current review.

### Eligibility criteria

Original studies in English or French were eligible for the review. Studies were screened on the basis of (a) clinical diagnosis of PTSD (by a clinician or validated instrument), (b) a case of PTSD acquired in a workplace environment, (c) the report of an intervention and its impact on clients, and (d) whether work outcomes were measured (i.e. return to work, work functioning).

Studies were excluded if the intervention was targeted specifically at another mental health condition rather than clients' PTSD symptoms (i.e. substance use disorders, major depressive disorder). Moreover, studies reporting on PTSD that was not acquired in the workplace also met criteria for exclusion. These included reports of PTSD following natural disasters, terrorist activity, and non-workplace violence or assault. Studies of combat-related PTSD were also excluded, since studies of treatment effectiveness in veterans show different responses to treatment compared to civilian samples [3]. In particular, veteran populations tend to be more resistant to first-time treatment, as a result of high comorbidity with substance use disorders, as well as the chronic nature of stressors encountered in a soldier's day-to-day work environment [3]. This makes the veteran population different from civilian working populations, since the latter do not typically face such high levels of stress as part of their long-term work routine. Moreover, veterans tend to have more access to structured psychosocial rehabilitation services, which are not widely used in civilian working populations [1]. Therefore, while populations like police officers may face similar stressors to soldiers, they do not have the same support systems in place to cope with resulting trauma. A final exclusion criterion was a diagnosis of secondary traumatic stress, which is a clinical designation for PTSD symptoms acquired vicariously through caring for trauma victims in a clinical setting.

After titles were screened, relevant abstracts were independently retrieved and screened for the same criteria. From these, full-text articles were evaluated based on the inclusion and exclusion criteria, and subsequently reviewed for quality. A third rater (CSD) performed an additional screening of 15% of titles and abstracts randomly selected from the list of 2522 titles. This was necessary as the inter-rater reliability was 0.71.

### Assessment of methodological quality

A 13-item quality assessment checklist was developed and piloted *a priori *for this review, modifying Lagerveld et al.'s checklist (2010) [19]. The quality assessment criterion as well as the scoring of each article reviewed is presented in Additional File 1. Checklist questions assessed study design, measurements of intervention, outcome, and data collection and analysis. Studies meeting all inclusion criteria were independently screened by AC and ES for quality. Any discrepancies in scoring were discussed, and a conclusion was reached through consensus. The mandatory quality criterion was the use of a comparison group. This comprised any form of control or waitlist group, or a multiple-measures design in which baseline measurements were compared with post-treatment outcomes and follow-up. Studies that failed to meet the mandatory quality criterion were excluded due to limited generalizability. For example, case studies, which do not include a comparison, were deemed insufficient evidence for the effectiveness of a given intervention. They were therefore excluded from the analysis at the quality assessment stage. Articles meeting all quality items were given a rating of excellent. Studies were rated as "good" if they did not meet all quality criteria, but received a passing score on the quality assessment measure (at least 6 out of 12).

## Results

**Figure 1:**
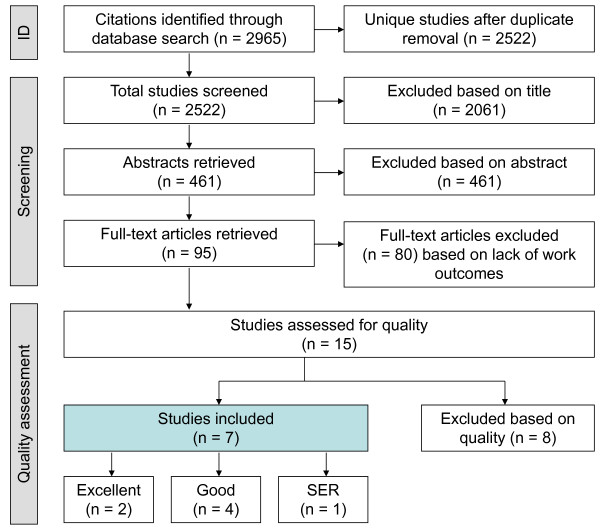
**Flowchart of literature search results and inclusion/exclusions**.

The systematic search through four databases (Medline, PsycINFO, Embase, and Web of Science) yielded a total of 2,965 publications. After removing 450 duplicates, two independent raters screened the remaining 2,522 titles. Of these, 95 full-text articles were reviewed based on title and abstract. Using the full-text versions, 80 articles were excluded mainly due to a lack of work outcomes, leaving a total of 15 studies for quality assessment. This process of inclusions and exclusions is depicted in Figure 1.

### Methodological quality

A total of 15 studies met all inclusion criteria, and were assessed for methodological quality. Of these, eight articles were case studies and were excluded from the current analysis on the basis of limited generalizability. Nevertheless, the results presented in these case studies are reviewed in the Discussion.

The remaining seven articles were included in the analysis, and were comprised of six research reports and one systematic review. Each of these papers was independently screened by ES and AC for quality assessment criteria adapted and developed *a priori*. The inter-rater reliability was 0.46. All disagreements in scoring were discussed and a conclusion was reached through consensus between the two independent raters. For example, if one rater did not award a particular study a point for a specific quality requirement, but this requirement was actually met and identified by the second rater, the score was changed to reflect this difference after discussion and consensus.

Upon review, all seven articles met criteria for good or excellent quality. The systematic review examined studies of interventions for workplace anxiety disorders, and included two studies of PTSD [11]. These studies were also retrieved in the present paper's literature search. Of the six research papers, two received a rating of excellent for meeting all quality criteria, and five were considered good.

### Characteristics of included studies

The six original studies included in the review covered a range of treatments and populations, and the main characteristics are presented in Table 2. Publication dates spanned from 1989 to 2008, with three studies published in 2000 or after. Participant populations included workers with occupation-related injuries (3 articles), police officers (1 article), and public transportation workers who had witnessed person-under-train incidents or had been assaulted at work (2 articles). The two papers examining transportation workers included part of the same study, and consisted of a report of post-treatment results, and a later report of work outcomes at 35-month follow-up. Treatment methods of the six articles included EMDR (2 articles), exposure-based treatment (3 articles), and brief eclectic psychotherapy (1 article), which is a combination of cognitive-behavioural and psychodynamic methods. Study location ranged from the Netherlands (1 study), to Sweden (2 studies), and the United States (3 studies). The six articles varied in study design, with three randomized controlled trials, and three pre-post outcome measure designs. All studies included return to work or full working capacity as a primary outcome measure. Summaries of intervention outcomes and main findings are presented in Table 3, and are discussed in more depth in the following results sections.

**Table 2:** Summary table of research article characteristics reviewed

Paper	Location	Population	Sample size	Study design	Treatment
Gersons et al., 2000	Netherlands	Police officers	42	RCT	Brief eclectic psychotherapy

Grunert et al., 1989	US	Injured workers	15	Pre-post, no control group	On-site work evaluations

Grunert et al., 1992	US	Injured workers	51	Pre-post, includes replication group	Graded work exposure

Högberg et al., 2006	Sweden	Public transportation workers	24	RCT	EMDR

Högberg et al., 2007	Sweden	Public transportation workers	20	RCT	EMDR

Weis, 1999	US	Injured workers	60	Pre-post	Prolonged imaginal exposure

WF = Work functioning

RTW = Return to work

EMDR = eye movement desensitization and reprocessing

**Table 3:** Summary table of intervention outcomes and main findings

Paper	Work outcome	Treatment	Follow-up period	Conclusion
Gersons et al., 2000	RTW	Brief eclectic psychotherapy	3 months	RTW in 86% of clients

Grunert et al., 1989	RTW	On-site work evaluations	12 months	RTW in 87% of clients

Grunert et al., 1992	RTW	Graded work exposure	6 months	RTW in 88% of clients

Högberg et al., 2006	WF	EMDR	Reported in Högberg et al., 2007	12 of 20 clients in treatment group no longer had PTSD post-treatment; improved WF

Högberg et al., 2007	RTW, WF	EMDR	35 months	RTW in 10 of the 12 remitters in Högberg et al. (2006)

Weis, 1999	RTW	Prolonged imaginal exposure	6 months	RTW in 83% of clients

RTW = Return to work

WF = work functioning

### Effects of EMDR on public transportation workers

Two articles assessed the effects of EMDR treatment on public transportation workers with PTSD. These articles followed the same group of participants, and followed the results of the same randomized controlled trial, at initial post-treatment stage and at 35-month follow-up respectively. The primary work outcome of this study was working capacity. The 12 participants in the initial experimental group received five 90-minute sessions of EMDR over two months, and were compared to 9 patients in the waitlist control group. The later follow-up study tested the long-term effects of EMDR in these initial 12 patients, plus 8 of the waitlist participants who did later undergo treatment. Results post-treatment showed that 12 of 20 clients in the treatment group no longer fulfilled the criteria for PTSD. At eight months, this figure was 14 of 20; at 35-month follow-up, 13 of 20 clients no longer had PTSD. Results from the treatment group showed a statistically significant difference to participants in the control group. With respect to work outcomes, 10 of the 12 clients who no longer had a diagnosis PTSD after treatment had returned to work with full working capacity at 35 months. Of those who retained the diagnosis, only 2 of 8 returned to work at 35 months. The results point to a possible association between PTSD symptoms and workers' ability to return to work with full functioning capacity.

### Effects of exposure-based treatment on employees injured at work

Three articles looked at exposure-based treatments for employees with occupation-related injuries and resulting PTSD. Particularly for work-injured clients, avoidance of the area where the injury occurred is a major barrier to recovery and work resumption [20]. All three studies measured return to work as the primary work outcome, and did not include a group of control participants (although they did include pre-post test and follow-up). In one study by Weis (1999), 83% of employees returned to work at 6-month follow-up [21]. Those who did not return to work showed little to no improvement in PTSD symptoms. Grunert et al. (1989) used on-site work evaluations as a form of environmental exposure for clients who had failed to benefit from traditional therapeutic methods [22]. In this study, 87% of clients returned to work within 8 weeks of the intervention, and maintained their employment status at 6- and 12-month follow-ups. Finally, a subsequent study by Grunert et al. (1992) examined the effect of graded work exposure as a form of desensitization to promote return to work in occupationally-injured workers [20]. Results showed that 92% of clients completed the treatment successfully, and 88% of these clients were still working at 6-month follow-up. Taken together, these studies suggest an average return-to-work rate of 85.05% at 6-month follow-up after exposure-based treatments.

### Effects of brief eclectic psychotherapy on police officers

One article examined the effects of brief eclectic psychotherapy -- a combination of psychodynamic and cognitive-behavioural approaches -- on a group of police officers with PTSD. This was a randomized control trial. Clients in the treatment group received weekly 60-minute individual psychotherapy sessions for 16 weeks. At baseline before treatment, 18% of clients in the treatment group had returned to work, while 59% had returned after 4 treatment sessions. At the end of treatment, 77% of experimental participants had returned to work, and 3-month follow-up measures indicated a return to work figure of 86%. The difference between return to work outcomes in experimental and control participants was only significant at 3-month follow-up.

### Add-on treatments for PTSD when regular psychotherapy fails

Interestingly, a subset of two studies addressed add-on treatments for workplace PTSD, which were used to compliment traditional psychotherapies like CBT or EMDR, or to treat clients who had failed to benefit from these treatments. In both cases, the use of an intervention specific to the workplace (on-site work evaluations and graded work exposure) was successful in promoting return to work (88% and 87% reported in each study). Both of these studies used a pre-post design for comparing initial symptoms to treatment outcome, but they did not include a control group.

### Assessment of follow-up

Macklin et al. (2000) has suggested that follow-up measurements of even 15 months are insufficient to determine the effects of treatment for PTSD, due to the chronic nature of the disorder [23]. Indeed, while a large body of research points to the effectiveness of CBT and EMDR for general PTSD, studies including follow-up periods longer than two years show less promising results, including inability to maintain treatment gains [23].

In the set of five studies included, only Högberg et al.'s (2008) group of participants was followed up at 35 months [24]. The study by Gersons et al. (2000) included a 3-month follow-up period, while Weis (1999) and Grunert et al. (1989, 1992) conducted follow-up at 6 or 12 months [25,21,8,20]. The effects of follow-up measurements on generalizability are discussed.

## Discussion

Interventions for work-related PTSD are critical for allowing employees to return to work so that they no longer experience the physical and psychological symptoms of stress, avoidance, and recurring trauma flashbacks. Taken together, the studies assessed in the current review offer increasing evidence for the bi-directional relationship between work status and PTSD symptoms. Those who did not return to work show little to no improvement in PTSD symptoms, while those who fail to reduce their PTSD symptoms have more difficulty returning to work [21,24]. Based on the studies reviewed, there is strong evidence that psychotherapy-based workplace interventions may be effective at improving employment outcomes for those with work-related PTSD. This conclusion is based on Ariens et al.'s (2000) assessment of levels of evidence for systematic reviews, which includes considerations of quality and the number high-quality studies in a given area of research [26].

Although there is strong evidence indicating the effectiveness of psychotherapy-based workplace interventions on improving employment outcomes, this systematic review was subject to publication bias. This was a result of searching four databases primarily containing peer-reviewed material, which retrieved few doctoral theses, and excluded conference material. Furthermore, due to a lack of fluency in languages other than English and French, papers in other languages were not considered.

One limitation within the papers analyzed is the heterogeneity of populations assessed. Clients in the present review varied in their number of exposures to traumatic events. This difference between single and multiple exposures can influence client symptoms, according to a cross sectional study by Hagenaars et al. (2011) [27]. Although it is unclear whether various symptom profiles respond differently to treatment, it is possible that complex cases of PTSD require more treatment sessions, or additional strategies to treat the disorder [28]. In the present review, the difference between multiple and single trauma exposures is important, because work-injured employees tended to have a single traumatic exposure, whereas police officers and public transportation workers often experience multiple traumatic events over the course of employment. Effective interventions for treating a single traumatic event at work may be very different from those targeting chronic exposure to trauma.

A further question of interest involves the various occupational cultures of these groups, as they relate to the chronicity of stressors at work. As Summerfield (2011) outlined in a report of sick absences in the police force, often the officers' decision to return to work was based on their own wish to return, rather than performance on clinical diagnostic tests [29]. Following health-related absences in the police force, officers may face the challenge of resuming their occupational roles in an environment where they feel little motivation, and may be more prone to ill health retirement [29]. The results of the present systematic review may be subject to variations among these specific occupational cultures. For example, the success of add-on treatments for PTSD when traditional psychotherapy fails could be a result of elements apart from the simple reduction of symptoms. Since these treatments involved on-site exposure to the workplace, they might also work toward reintegrating workers into the context and culture of the workplace. In other words, treatments that focus beyond simply desensitizing patients to reminders of the trauma may have the most success because they provide motivation for workers, so that they in fact want to return to work.

The present review is also limited by its small pool of articles, which, in itself, points to the lack of research on interventions for work-related PTSD. Among the common limitations of studies reviewed was the lack of long-term follow-up, with most studies not extending past 12 months. Moreover, the small sample sizes found in the studies suggests limited generalizability to the population of all workers with PTSD, especially due to the samples recruited. For instance, Gersons et al. (2000) studied police officers who had been referred to their study by an occupational physician -- which introduces a possible selection bias and potential for an unrepresentative sample [25]. The authors recognized this limitation, and explained that their participants constituted a treatment-seeking group that was highly motivated for treatment. They acknowledged that this does not necessarily represent the population of police officers at large.

Apart from the original studies retrieved by the systematic review of the literature, the search also produced eight case studies. The dates of publication ranged from 1991 to 2005, with four studies published after 2000. Case studies still form a significant portion of this growing body of research, and point to the need for more extensive controlled studies to determine the most effective treatments for PTSD. The eight case studies used a range of treatments to treat PTSD in workers. Specifically, two studies used EMDR therapy, four examined cognitive-behavioural or exposure-based interventions, one studied a combination of hypnosis and cognitive-behavioural methods, and one looked at a cognitive restructuring therapy component as adjunctive treatment for when prolonged exposure fails to relieve PTSD symptoms in workers. Populations of these studies included injured workers, police officers, fire fighters, and store clerks.

## Conclusion

The current state of the literature indicates the need for further research on the topic of work-related PTSD, and on the best strategies for improving work outcomes. As it stands, there is promising evidence for the effectiveness of interventions targeted at workers with PTSD; however, in populations such as those injured at work, traditional psychotherapy is at times ineffective [8,20]. More work on the use of add-on therapies and how to adapt them to specific work settings may therefore offer a solution tailored to the workplace for improving symptoms and work outcomes.

## Competing interests

The authors declare that they have no competing interests.

## Authors' contributions

ES led the conception, design, data acquisition, analysis and interpretation of the data. AC collaborated on the design, data acquisition and analysis. CC collaborated on the design and acquisition of data. SB collaborated on the design and data acquisition. CSD collaborated on the conception, design and acquisition of data, and supervised the data analysis and interpretation. All authors read and approved the final manuscript.

## APPENDIX 1: Search strategy

**Database: **Medline

Search Terms:

(exp Occupational Health or exp Occupational Medicine or exp Occupational Diseases or exp Employment or exp Work or exp Occupational health*.mp. or exp Occupational intervene*.mp. or exp Occupational therap*.mp. or Vocational rehab*.mp. or Occupat* diseas*.mp. or Workplace*.mp. or Work place*.mp. Work.mp. or Employ*.mp. or Return to work.mp. or Absent*.mp. or Sick leave.mp. or Sick* absen*.mp. or Job.mp. or Jobs.mp.) and (exp Stress Disorders, Traumatic or PTSD*.mp. or Post traum* stress*.mp. or Stress post* traum*.mp.) and (exp Patient Care or exp Drug Therapy or exp Psychotherapy or exp Treatment Outcome or (Treat*.mp. or Interven*.mp. or CBT*.mp. or cognitive behav* therap*.mp or Group therap*.mp. or Psychother*.mp. or Psych* interven*.mp. or Drug therap*.mp. or Pharmacother*.mp. or Complementary and alternative medicine*.mp. or Meditat*.mp)

**Database: **PsycINFO

Search Terms:

(exp Employee Attitudes or exp Work (Attitudes Toward) or exp Occupational Stress or exp Employee Absenteeism or exp Employee Assistance Programs or exp Occupational Health or exp Occupational Exposure or Occupational interven*.mp. or Occupational therap*.mp. or Vocational rehab*.mp. or Occupat* diseas*.mp. or Workplace*.mp. or Work place*.mp. or Work.mp. or Employ*.mp. or Return to work.mp. or Absent*.mp. or Sick leave.mp. or Sick* absen*.mp. or Job.mp. or Jobs.mp.)

and (exp Posttraumatic Stress Disorder or exp Emotional Trauma or PTSD.mp. or Post traum* stress*.mp. or Stress post* traum*.mp.) and (exp Treatment Treatment Outcomes or exp Intervention or Treat*.mp. or Therap*.mp. or Interven*.mp. or CBT*.mp. or Cognitive behav* therap*.mp. or Group therap*.mp. or Psychotherap*.mp or Psych* interven*.mp. or Drug therap*.mp. or Pharmacother*.mp or Complementary and alternative medicine*.mp. or Meditat*.mp.)

**Database: **Web of Science

Search Terms:

(Occupational exposure or Absentee* or Absenteeism or Sick* absence or Sick leave or Return to work or Workplace or Work place or Vocational rehabilitation or Occupational therap* or Occupational intervent* or Employ* or Occupational disease* or Occupational medicine or Occupational health) and (PTSD or Posttraumatic stress disorder or PTSD or Post traum* stress* or Stress post* traum*) and (Treat* or Treatment outcome* or Psychotherap* or Drug therap* or Patient care or Meditat* or Holistic medicine or Complementary alternative medicine or Pharmacotherap* or Psych* interven* or Cognitive behav* therap* or CBT or Interven*)

**Database: **Embase

Search Terms:

(exp Occupational health or exp Occupational medicine or exp Occupational disease or exp Employment or exp Employee attitude or exp Employee or exp Job stress or exp Absenteeism or exp Job performance or exp Productivity or Workplace* or Occupational health*.mp. or occupational interven*.mp. or occupational therap*.mp. or vocational rehab* .mp. or occupat* diseas*.mp. or workplace*.mp. or work place*.mp. or employment*.mp. or return to work.mp. or absentee*.mp. or sick leave.mp. or sick* absenc*.mp.) and (exp Posttraumatic stress disorder or PTSD.mp. or post traum* stress*.mp. or stress post* traum*.mp.) and (Psychiatric treatment or exp Drug therapy or exp Clinical study or exp Treatment outcome or exp Therapy treat*.mp. or interven*.mp. or CBT*.mp. or cognitive behav* therap*.mp. or group therap*.mp. or psychotherap*.mp. or psych* interven*.mp. or drug therap*.mp. or pharmacotherap*.mp. or complementary alternative medicine.mp.or meditat*.mp.)

## Pre-publication history

The pre-publication history for this paper can be accessed here:

http://www.biomedcentral.com/1471-2458/11/838/prepub

## Supplementary Material

Additional file 1 **Quality assessment checklist**. The additional file contains the quality checklist criterion that was used to determine the quality of the papers being analyzed for the systematic review. Scores of each article are displayed as well as the quality checklist items that were adapted from Lagerveld et al. (2010). Click here for file
