# Supplementary material for: Interventions to improve work outcomes in work-related PTSD: a systematic review
Source: BMC Public Health. 2011 Oct 31;11:838. doi: 10.1186/1471-2458-11-838 (PMC3219578; doi:10.1186/1471-2458-11-838)
Supplement: Additional file 1 — Quality assessment checklist. The additional file contains the quality checklist criterion that was used to determine the quality of the papers being analyzed for the systematic review. Scores of each article are displayed as well as the quality checklist items that were adapted from Lagerveld et al. (2010). [file 1471-2458-11-838-S1.DOC]

Quality Assessment Checklist

| **Paper** | **Work outcome** | **1** | **2** | **3** | **4** | **5** | **6** | **7** | **8** | **9** | **10** | **11** | **12** | **13** | **TOTAL** |
| --- | --- | --- | --- | --- | --- | --- | --- | --- | --- | --- | --- | --- | --- | --- | --- |
| Grunert (1989) | RTW | Yes | 1 | 0 | 0 | 1 | 1 | 1 | 1 | 0 | 0 | 1 | 1 | 0 | 7 |
| Gersons (2000) | RTW | Yes | 1 | 1 | 1 | 1 | 1 | 1 | 1 | 1 | 1 | 1 | 1 | 1 | 12 |
| Högberg (2007) | RTW, WF | Yes | 1 | 1 | 1 | 1 | 1 | 0 | 1 | 1 | 1 | 1 | 1 | 1 | 11 |
| Högberg (2008) | RTW, WF | Yes | 1 | 1 | 1 | 1 | 1 | 1 | 1 | 1 | 1 | 1 | 1 | 1 | 12 |
| Grunert (1992) | RTW, WF | Yes | 1 | 1 | 1 | 1 | 1 | 1 | 1 | 0 | 0 | 1 | 1 | 0 | 9 |
| Noordik (2010) | RTW, WF | SER | 1 | 1 | n/a | 1 | 1 | 1 | n/a | n/a | 1 | 1 | 1 | 1 | 9 |
| Weis (1999) | RTW | Yes | 1 | 1 | 0 | 1 | 1 | 1 | 1 | 1 | 1 | 1 | 1 | 1 | 11 |

Quality Assessment Criterion:

1 = Includes comparison group similar to experimental group (if no, exclude article)

2 = clearly describes intervention

3 = States main features of the population studied (Sex, age, location of recruitment)

4 = Mentions initial participation rates

5 = States method of data collection

6 = States work status of subjects pre-treatment

7 = States work outcomes post-treatment
8 = States attrition rates at each measurement point of study

9 = Mentions and accounts for confounds

10 = Uses statistical model that is appropriate to the outcome measured

11 = Answers initial research question

12 = Discusses study limitations (and biases) and implications for study conclusion

13 = Tests statistical significance of the association, presenting relevant parameters
